# Complete Recovery of *Acanthamoeba* Motility among Surviving Organisms after Contact Lens Care Disinfection

**DOI:** 10.3390/microorganisms11020299

**Published:** 2023-01-23

**Authors:** Allison Campolo, Brian Patterson, Esther Lara, Paul Shannon, Monica Crary

**Affiliations:** Alcon Research, LLC, Fort Worth, TX 76134, USA

**Keywords:** *Acanthamoeba*, contact lenses, contact lens solution, motility, microbial keratitis

## Abstract

*Acanthamoeba* keratitis is a sight-threatening infection of the cornea which is extremely challenging to treat. Understanding this organism’s responses during contact lens contact and disinfection could enhance our understanding of how *Acanthamoebae* colonize contact lens cases, better inform us on contact lens care solution (CLC) efficacy, and help us better understand the efficacy required of CLC products. To explore this gap in knowledge, we used *Acanthamoeba* ATCC 30461 and ATCC 50370 trophozoites to examine *Acanthamoeba* behavior during and after CLC disinfection. Amoebae were added to sterile aluminum flow cells and flow cell solutions were changed to Ringer’s solution (control), or one of four CLCs based on biocides (PHMB, PAPB/Polyquad, Polyquad/Aldox, or Polyquad/Alexidine) for 6 h. Each flow cell solution was then changed to axenic culture media (AC6) for 12 h to determine the behavior of amoebae following disinfection. Distance, speed, and displacement were calculated for each organism. As compared to the control of one-quarter Ringer’s solution, each CLC significantly impacted *Acanthamoeba* motility in both the CLC and AC6 conditions. However, the amoebae challenged with the PHMB CLC traveled a significantly greater total distance than with the other three CLCs, indicating differences in effectiveness between biocides. Furthermore, amoebae regaining motility post-disinfection by CLCs were observed to travel considerable distances and thus could be considered dangerous to ocular health. We determined that while all CLCs produced a substantial or complete cessation of movement vs. the control condition during disinfection, those which relied on the Polyquad biocides were the most effective, and that any amoebae which survived disinfection were able to recover motility. Future examinations of these findings should include direct correlations between motility and viability, and how infectivity and motility may be related.

## 1. Introduction

*Acanthamoeba* keratitis (AK) is a parasitic infection that follows the attachment of *Acanthamoeba*, and is often the result of contact lenses becoming contaminated with water, from sources such as tap water, lakes, and streams [1,2]. As a result, AK is typically the result of poor contact lens hygiene practices, such as failing to rinse and rub lenses, topping off of contact lens care solutions (CLCs) instead of replacing solutions daily, wearing lenses while bathing or swimming, or rinsing lenses in tap water [3,4]. Furthermore, outbreaks of AK can be attributed to ineffective CLCs which promote encystment or are unable to disinfect trophozoites [5,6]. Therefore, it is critical to understand how CLCs affect *Acanthamoebae* when used for the time periods stated in the manufacturer’s instructions. Currently, there are no requirements to demonstrate *Acanthamoeba* disinfection efficacy for any product on the market, although there is a call for the International Standards Organization to examine the protocols necessary for such a guideline, for commercially-produced CLCs [7]. 

Overall, there is a lack of research and understanding regarding *Acanthamoeba* motility and how it relates to contact lens use, or how it is impacted by CLC disinfection. We recently published a novel method regarding *Acanthamoeba* motility on control surfaces with and without available nutrients [8], and found that amoebae were able to remain motile for at least 12 h even in the absence of nutrients [9]. This indicated that amoebae could potentially travel substantial distances on contaminated lenses, or from a lens to a cornea and into the corneal epithelium, within the normal period of time for either wearing a lens or in which the lens is in its case. It remains to be determined how motility is affected by CLCs, and if that motility is recoverable following the disinfection period. Therefore, this study seeks to demonstrate the strengths and weaknesses of common commercially-available CLCs, using quantitative time-lapse image analysis to assess *Acanthamoeba* movement. By understanding how biocides and CLC composition may affect amoeba motility, further efforts can be made to develop the best products for patients. Future research built on the data presented here, may additionally further understanding of how motility and speed (particularly post disinfection) may affect infectivity, allowing industrial and clinical standards to be sharpened in the effort to provide the highest levels of patient safety.

## 2. Materials and Methods

### 2.1. Acanthamoeba Trophozoite Culturing, and Contact Lens Care Solutions Used

As previously described [8,10], trophozoites were axenically cultured in AC6 medium (axenic culture medium; containing 20 g biosate peptone, 5 g glucose, 0.3 g KH_2_PO_4_, 10 µg vitamin B12, and 5 mg L-methionine per liter of distilled deionized water). AC6 was adjusted to pH 6.6–6.95 with 1M NaOH and autoclaved at 121 °C for 20 min, before being stored at room temperature for use within 3 months. Organisms were harvested using one-quarter Ringer’s solution. *Acanthamoeba* strains were obtained from American Type Culture Collection (ATCC, Manassas, VA, USA). *Acanthamoeba polyphaga* (ATCC 30461), Group T4, isolated from a human eye infection (Namibia or South Africa, 1973) and *Acanthamoeba castellanii* (ATCC 50370), also Group T4, isolated from a human eye infection (New York, NY, 1978) were the two clinical strains used in this study. Importantly, these two commonly used clinically relevant strains belong to the T4 genotype, which is the genotype most frequently associated with *Acanthamoeba* keratitis [11,12,13]. To create a homogenous population of *Acanthamoeba* trophozoites, amoebae were scaled up in fresh AC6 media 24 h prior to testing. The multi-purpose solutions tested were chosen for their representation of popular multi-purpose solutions, are identified by biocide throughout the manuscript, and can be found in Table 1. 

In order to simulate the common practice of patients of re-using old solutions, cycled PHMB was created by filling the manufacturer-provided contact lens cases with PHMB. Lenses were placed in cases overnight for 12 to 16 h and then removed. This was repeated for 7 days without replacing or refreshing the CLC, and the resulting CLC was collected. 

### 2.2. Acanthamoeba Flow Cell Suspension

**Figure 1:**
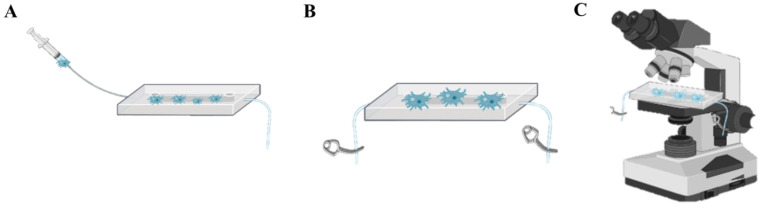
Schematic representation of flow cell apparatus. (**A**) *Acanthamoebae* are added to the flow cell apparatus. (**B**) Once amoebae have filled the flow cell, the ports are clamped to prevent further fluidic movement. (**C**) The flow cell is attached to the microscope stage for the duration of the experiment to prevent physical interference with *Acanthamoeba* motility.

Similar to our previous description [8], sterile aluminum transmission flow cells (Biosurface Technologies Corporation, Bozeman, MT, USA) were assembled with glass coverslips (Figure 1). *Acanthamoeba* suspensions (3.0 × 10^4^ cells/mL) in one-quarter Ringer’s solution were added through the ports of the flow cells. Each flow cell was placed on the microscope stage and kept there for the duration of the experiment, to reduce physical interference with *Acanthamoeba* behavior. The flow cell ports were clamped shut to stop the flow of the *Acanthamoeba* suspension, and *Acanthamoeba* trophozoites were allowed to adhere to glass coverslips for one hour. Following this, flow cell solutions were either maintained as one-quarter Ringer’s solution, or changed to one of the four contact lens care solutions (CLCs). To exchange fluids, 4 mL of a solution was slowly added through the ports of the flow cell, taking care not to disturb the adhered amoebae. The flow cell ports were again clamped to prevent fluidic movement, and amoebae were imaged for 6 h (6 h was used to create identical testing conditions, as the majority of the solutions used specify a 6-h disinfection time, although PAPB/PQ requires only 4 h). After 6 h, all solutions were exchanged for axenic culture media (AC6) to determine the ability of *Acanthamoebae* to recover following disinfection. The AC6 condition was imaged for a further 12 h, for a total experimental imaging time of 18 h. Images were taken at 4× magnification using a Nikon Eclipse Ti-U Microscope (Nikon, Tokyo, Japan), every 24 s. The images were subsequently combined into a video format using NIS Elements AR 3.2. 

### 2.3. Acanthamoeba Track Analysis

**Figure 2:**
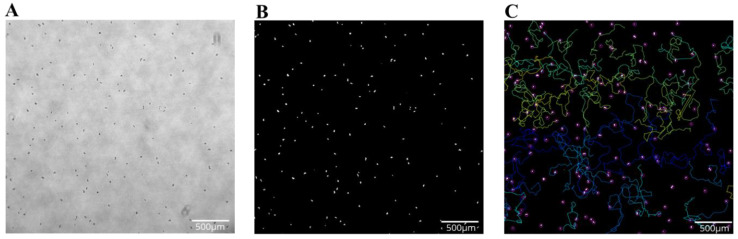
Representative images of the track analysis process. (**A**) Bright-field microscope image taken in original grayscale. (**B**) Same image converted to binary, where *Acanthamoebae* appear as white organisms on a black background. (**C**) Same image with selected tracks shown (not all tracks present), where ImageJ has assigned a unique color for each individual track. Magnification = 4×, scale bar = 500 um.

Videos were recorded in grayscale using bright-field microscopy (Figure 2). Using ImageJ, videos were converted into a high-contrast, binary format for analysis. As previously reported, background was subtracted by setting the rolling ball to 50.0, and the contrast was enhanced by 0.1%. Automatic thresholding, as determined by ImageJ, was used to convert grayscale images into binary images. Non-amoebic artifacts were removed by utilizing the fill and clear functions as required. Binary videos were then analyzed using ImageJ Trackmate software to quantify amoeba movement, using an estimated blob size of 35 µm, and allowing for 40 µm of movement between frames. Trackmate was set to allow 4 frame gap closures if movement was less than 100 µm for the identified cell, and tracks representing less than 95% of the frames captured were discarded. Three independent replicates were recorded for each condition per strain, with between 20 and 200 trackable amoebae in each replicate. Tracks were considered unusable if amoebae walked on or off of the field of view at any time during recording. Total distance, max distance, speed, and displacement were calculated for each organism track in a replicate. Within the 18-h experimental period, videos were divided into one-hour sections consisting of 151 frames each, and the data over that time period was averaged. 

### 2.4. Tracking Algorithms

**Figure 3:**
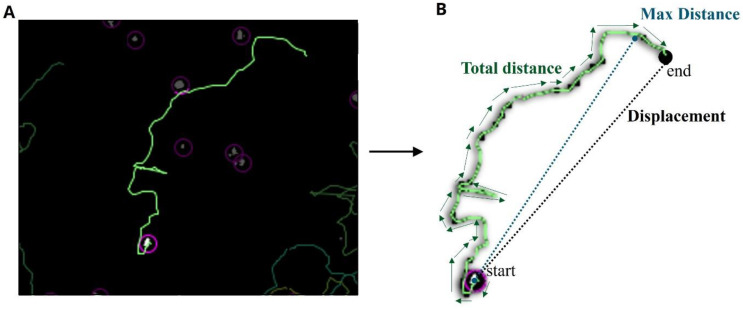
Representative image of track algorithms. (**A**) Original tracked image. (**B**) Representative parameters describing how total distance, max distance, and displacement are determined.

As previously described [8], the parameters were calculated and are visually depicted in Figure 3. Total distance traveled is the total µm an amoeba walked over any one-hour period. It is calculated as

$total\;distance\;traveled=\overset{}{\underset{i}{\sum }}d_{i,i}+1$ where *d_i,i_* + 1 is the distance from one spot to the next spot in the track. 

Max distance traveled represents the distance to the furthest point of the track, with respect to the first spot of the track. This is computed by finding the straight-line distance between these two points. This is calculated as 

$max\;distance\;traveled=Max_{i.j}\left(d_{ij}\right)$ where *d_ij_* is the distance from any spot *i* to any spot *j* in the track.

Mean speed (not to be confused with mean straight-line speed) is defined as the total distance divided by the total track time. This is calculated as 

$mean\;speed=\frac{total\;distance}{total\;track\;time}$ to determine the number of microns traveled per second. 

Finally, displacement is the measured distance between the last spot of the track and the first spot of the track in time.

### 2.5. Statistics

The data from each amoeba track within a replicate were averaged, and then each replicate averaged for a sample size of 3 for each time point, condition, and strain. To accurately compare the average distances traveled between conditions, within any one time point the average distance moved by amoeba in that time point, was multiplied by the number of moving amoebae in that frameset, resulting in a number which gave the net total number of microns moved for that time period (Supplemental Video S1). Statistical analysis was completed via two-way repeat measures ANOVA for comparison between strains within each condition and time point, and within each strain’s own baseline. This comparison was followed by Tukey’s post hoc multiple comparisons test and significance was set at *p* < 0.05. Due to the high number of comparisons per graph and to maintain legibility of each graph, *p*-values for between-condition comparisons are displayed graphically as subpanels in the associated figures. *p*-values were calculated to eight decimal points to visualize differences between conditions. Every value above 0.99999999 or below 1 × 10^−8^ is displayed as 0.99999999 or 1 × 10^−8^, respectively. 

## 3. Results

Amoebae were challenged with disinfecting CLCs or one-quarter Ringer’s solution for 6 h, and the solution was subsequently changed to AC6 media for the following 12 h to understand the capability of the surviving amoebae to recover from disinfection. The resulting data, displayed in relative units (RU), were calculated by multiplying the average distance moved by amoebae, by the number of moving amoebae, to give the total number of microns moved in any timeframe (Video S1). The percentage of moving amoebae in any field of view (as opposed to those which were non-motile), as a subset of the total amoebae in the field in any condition, is noted in Figure S1. 

**Figure 4:**
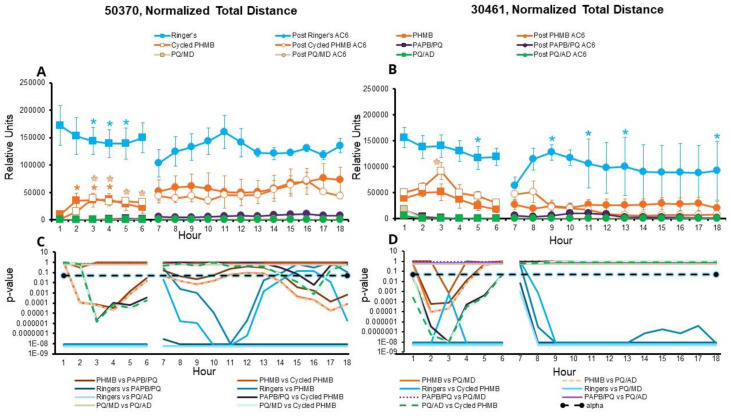
Surviving *Acanthamoebae* recover total distance traveled after disinfection. Amoebae were disinfected or put in one-quarter Ringer’s solution for the first 6 h, followed by AC6 media for the following 12 h (total 18 h). Normalized total distance is presented as mean ± SE. (**A**) *Acanthamoeba castellanii* (ATCC 50370), and (**B**) *Acanthamoeba polyphaga* (ATCC 30461). Relative units: average total distance moved by each amoeba in the field of view, multiplied by the number of moving amoebae, resulting in the total number of microns moved by all amoebae. * *p* < 0.05 vs. each condition’s own baseline (either hour 1 or hour 7 for the CLC or AC6 conditions, respectively). Statistical comparisons between conditions are represented in (**C**) *p*-values for (**A**), and (**D**) *p*-values for (**B**). Alpha was set at 0.05. *n* = 3/group.

When examining the total distance traveled, amoebae in the one-quarter Ringer’s solution traveled significantly farther in any hour compared to those in the CLCs in both amoeba strains tested (*p* < 0.05, Figure 4, Figure S2A,B), as expected, with a few exceptions being noted for comparisons between one-quarter Ringer’s solution and either PHMB or cycled PHMB. Other significant results include: 

Both ATCC 50370 and ATCC 30461 strains traveled a significantly farther total distance when challenged with PHMB than when challenged with PQ/AD, PQ/MD, and PAPB/PQ (all polyquad-based solutions), in the first 6 h. 

With the ATCC 50370 strain, this was similarly true for the subsequent 12 h after the media had been changed to AC6. 

With the ATCC 50370 strain, amoebae in both PHMB and cycled PHMB traveled significantly farther at later time points than at their hour 1 CLC baseline. 

Trophozoites exposed to cycled PHMB maintained a significantly greater distance than those exposed to all PQ solutions, for hours 3 through 6 in the ATCC 50370 strain, and hours 1 through 6 in the ATCC 30461 strain. 

With the ATCC 30461 strain, amoebae in cycled PHMB traveled a significantly greater distance than those in PHMB, during hour 3. 

At no point were the effects of PQ/AD or PQ/MD significantly different from each other, and their results were so similar in every experiment that their significance lines often overlapped (Figure 1C,D). 

**Figure 5:**
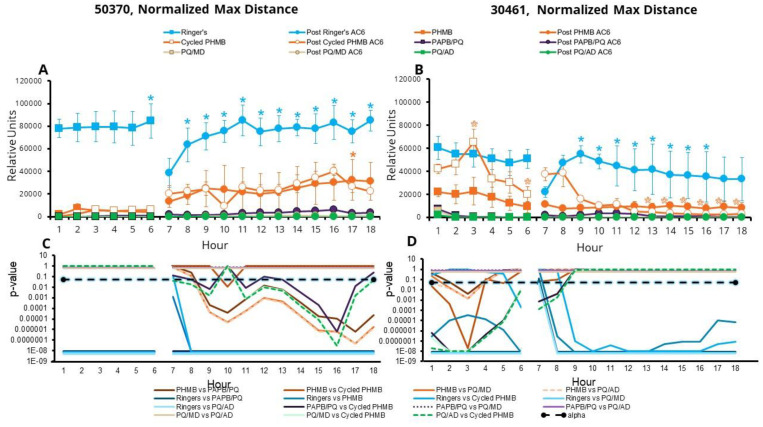
Surviving *Acanthamoebae* recover max distance traveled after disinfection. Amoebae were disinfected or put in one-quarter Ringer’s solution for the first 6 h, followed by AC6 media for the following 12 h (total 18 h). Normalized max distance is presented as mean ± SE. (**A**) *Acanthamoeba castellanii* (ATCC 50370), and (**B**) *Acanthamoeba polyphaga* (ATCC 30461). Relative units: average maximum distance moved by each amoeba in the field of view, multiplied by the number of moving amoebae, resulting in the total number of microns moved by all amoebae. * *p* < 0.05 vs. each condition’s own baseline (either hour 1 or hour 7 for CLC or AC6 conditions, respectively). Statistical comparisons between conditions are represented in (**C**) *p*-values for (**A**), and (**D**) *p*-values for (**B**). Alpha was set at 0.05. *n* = 3/group.

Similar results were observed for the max distance traveled (Figure 5), with the notable exceptions that all solutions resulted in less distance traveled in the first 6 h, compared to one-quarter Ringer’s solution for the ATCC 50370 strain, and all solutions resulted in a similar distance traveled in the first 6 h, compared to PHMB and cycled PHMB for the ATCC 50370 strain. Other significant results included: 

With both the ATCC 50370 and ATCC 30461 strains, at hour 7 the difference of max distance of PHMB and Cycled PHMB relative to one-quarter Ringer’s solution was less than the difference at hour 6 and there were no significant differences, and with the ATCC 30461 strain, the effect of PAPB/PQ was not significantly different from that of one-quarter Ringer’s solution. 

In both PHMB and cycled PHMB, the ATCC 50370 strain demonstrated significantly greater movement than in all PQ solutions, starting at hour 9 and maintaining this difference until the end of the experiment.

In PHMB, the ATCC 50370 strain moved significantly farther at hour 17 vs. its hour 7 baseline in the AC6 condition, suggesting a substantial recovery post disinfection. 

With the ATCC 30461 strain, amoebae in cycled PHMB moved significantly more than those in PHMB for hours 1 through 3, and more than those in all PQ solutions for hours 1 through 8. 

There were no significant differences among all PQ solutions with either strain. 

Although the two *Acanthamoeba* strains are not being directly compared, it is interesting to note that it is during the second 12 h (the AC6 portion) of the experiment when the CLC-to-CLC statistical differences are apparent regarding max distance for the ATCC 50370 strain, while with the ATCC 30461 strain, the majority of the CLC-to-CLC statistical differences are found in the first 6 h. 

**Figure 6:**
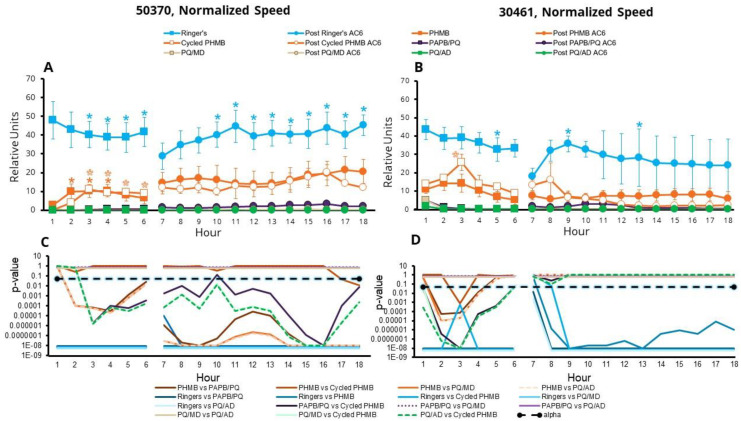
Surviving *Acanthamoebae* recover speed after disinfection. Amoebae were disinfected or put in one-quarter Ringer’s solution for the first 6 h, followed by AC6 media for the following 12 h (total 18 h). Speed is presented as mean ± SE µm per second, multiplied by the number of moving amoebae per field of view (relative units). (**A**) *Acanthamoeba castellanii* (ATCC 50370), and (**B**) *Acanthamoeba polyphaga* (ATCC 30461). * *p* < 0.05 vs. each condition’s own baseline (either hour 1 or hour 7 for CLC or AC6 conditions, respectively). Statistical comparisons between conditions are represented in (**C**) *p*-values for (**A**), and (**D**) *p*-values for (**B**). Alpha was set at 0.05. *n* = 3/group.

Speed (Figure 6, Figure S2C,D) was calculated as microns per second and multiplied by the number of moving amoebae. Statistically, speed was extremely similar to max distance, with the exception that there were no differences in the speed of moving amoebae compared to one-quarter Ringer’s solution in the first six hours with the ATCC 50370 strain. Other significant results include:

In the 12 h of AC6 recovery, ATCC 50370 trophozoites that had been exposed to PHMB were significantly different to amoebae treated with every other CLC and one-quarter Ringer’s solution, and those exposed to PHMB were slightly faster than those exposed to cycled PHMB at hour 18. 

ATCC 50370 amoebae in one-quarter Ringer’s solution were also faster than those in every CLC during the 12 h AC6 recovery period. 

For both strains, the effects of the PQ solutions were not significantly different from one another, regarding speed, at any time. 

With the ATCC 30461 strain, amoebae in PHMB were significantly slower than those in cycled PHMB at hour 3 (during the active disinfection phase). 

With the ATCC 30461 strain, amoebae in PHMB were faster than those in PQ solutions, for hours 1 through 4. 

In both strains, amoebae in cycled PHMB were significantly faster than those in PQ solutions, for hours 1 through 6. 

On a related note, ATCC 50370 amoebae in cycled PHMB moved significantly faster relative to the hour 1 CLC baseline, for hours 3 through 6, suggesting poor disinfection efficacy of topped-off CLCs. 

**Figure 7:**
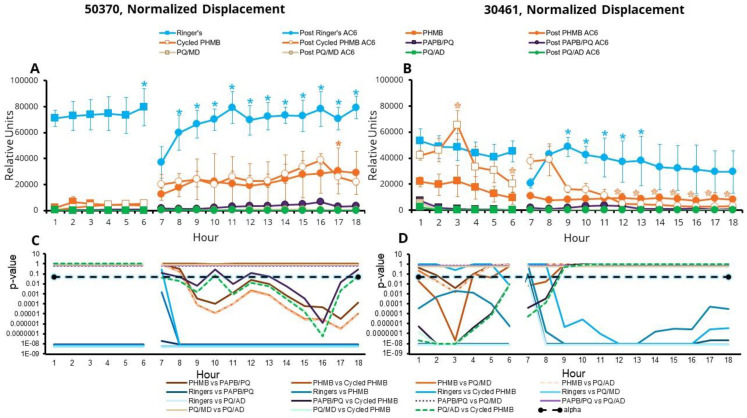
Surviving *Acanthamoebae* recover displacement after disinfection. Amoebae were disinfected or put in one-quarter Ringer’s solution for the first 6 h, followed by AC6 media for the following 12 h (total 18 h). Normalized displacement is presented as mean ± SE. (**A**) *Acanthamoeba castellanii* (ATCC 50370), and (**B)** *Acanthamoeba polyphaga* (ATCC 30461). Relative units: average displacement of each amoeba in the field of view, multiplied by the number of moving amoebae, resulting in the total number of microns moved by all amoebae. * *p* < 0.05 vs. each condition’s own baseline (either hour 1 or hour 7 for CLC or AC6 conditions, respectively). Statistical comparisons between conditions are represented in (**C**) *p*-values for (**A**), and (**D**) *p*-values for (**B**). Alpha was set at 0.05. *n* = 3/group.

Finally, displacement, that is, the distance between an amoeba’s start and end points, indicating the circularity of the path, was calculated (Figure 7). Significant results include: 

With the ATCC 50370 strain, in one-quarter Ringer’s solution there was significantly greater displacement than in all CLCs throughout the experiment, except for in cycled PHMB at hour 7. 

With the ATCC 50370 strain, no CLCs resulted in significantly different displacement to other CLCs, for hours 1 through 6. 

With the ATCC 50370 strain, the effects of PHMB and cycled PHMB were not significantly different from one another. However, for hours 9 through 18, all PQ solutions had significantly different effects than both PHMB and cycled PHMB, with the exception of a few hours for PAPB/PQ vs. cycled PHMB. 

With the ATCC 30461 strain, not only was the effect of cycled PHMB not statistically different from that of one-quarter Ringer’s solution in the first 5 h, but cycled PHMB actually resulted in a slightly greater displacement. 

With the ATCC 30461 strain, PHMB resulted in significantly greater displacement than cycled PHMB, for hours 1 through 4. 

With the ATCC 30461 strain, all PQ solutions resulted in significantly greater displacement than cycled PHMB, for hours 1 through 9. 

With the ATCC 30461 strain, all PQ solutions resulted in greater displacement than PHMB for hours 2 and 3. The effect of one-quarter Ringer’s solution was not different from that of PAPB/PQ or PHMB, at hour 7 only. 

ATCC 30461 amoebae exposed to PHMB moved significantly farther at the hour 17 time point compared to the hour 7 AC6 recovery baseline, again indicating a substantial recovery of motility following disinfection. 

There were no differences in the effects of any of the PQ solutions at any time.

## 4. Discussion

As evidenced by the increase in the number of investigations into, and publications on *Acanthamoeba* keratitis in recent years [5,6,8,10,14,15,16,17,18,19,20,21,22], this is a devastating and potentially sight-threatening disease. However, until now there has been a lack of investigation regarding the behavior of *Acanthamoeba* on contact lenses, both in and out of contact lens care disinfecting solutions (CLCs). We recently demonstrated that *Acanthamoebae* are able to maintain constant motility on a variety of surfaces, both with and without nutrients, for at least 12 h, and we also demonstrated new ways to quantify amoeba movement [8]. Following this, the question becomes how does CLC disinfection alter those natural movements, as there has been a lack of real-time analysis of how disinfection affects *Acanthamoeba* behavior and motility. Most importantly, it was unknown if *Acanthamoeba* recovers from disinfection and how disinfection may ultimately affect motility. Specifically, although it has been previously reported that an extremely small number of amoebae may produce a devastating *Acanthamoeba* infection [23], it was unknown if the amoebae which survive disinfection are as motile—and therefore could be as dangerous to the cornea—as amoebae before disinfection. Understanding this difference could clarify the necessity for products that result in complete disinfection, in order to keep patients safe. 

Here, we quantified the total distance traveled by amoebae during and after disinfection, as well as the max distance (the space between its starting point and furthest point traveled), speed, and displacement (the distance between the starting and ending points). These metrics indicate not only the speed on an hour to hour basis, but also estimate directionality or circularity of movement (whether an amoeba moves in a straight line from its starting point, or stays close to its point of origin). Essentially, these metrics could tell us (speculatively, as these motility studies have not been performed on opaque surfaces or in vivo) if amoebae can travel the distance from a lens case to a lens, or a lens to a cornea, or a corneal epithelium to a Bowman’s membrane or to a Descemet’s membrane. The human cornea is roughly 540 µm thick; in real terms, the amoebae in this study were able to travel hundreds of µm per hour in one-quarter Ringer’s solution, 90 µm per hour during the disinfection period (surviving and motile amoebae) and hundreds of µm per hour post disinfection (surviving and motile amoebae), unfortunately meaning that they can traverse a significant distance, either during or after the disinfection period. The only way to guarantee safety is to demonstrate no movement whatsoever during both the CLC and following AC6 conditions, which suggests cell death, as PQ/MD and PQ/AD were able to achieve. 

Several critical observations were prominent from our findings. Firstly, as is demonstrated clearly, some amoebae may cease movement during disinfection but then go on to be both alive and motile once they have recovered in the AC6 condition. Considering that this recovery media contains only supportive nutrients and no edible bacteria to sustain recovery (and this is used after amoebae have been shifted through the changing osmolarities of the culture medium to one-quarter Ringer’s solution, then to a CLC and then back to AC6), this is a jarring finding, demonstrating substantial and robust recovery following disinfection. Next, while the two strains studied here were not statistically compared, we did note that they demonstrated similar findings regarding one-quarter Ringer’s solution vs. all other CLCs, and regarding PHMB or cycled PHMB relative to all other CLCs. That is, while in one-quarter Ringer’s solution amoebae predictably demonstrated the maximum amount of movement, the effects of PHMB and cycled PHMB were very similar to those of one-quarter Ringer’s solution regarding speed, distance traveled, and pattern of movement, at many time points. This is a strong indication that PHMB alone (at a concentration of 0.0001%) does not demonstrate a high enough disinfection efficacy to be safe against *Acanthamoeba* trophozoites, as is demonstrated by their continued motility during and after PHMB disinfection. Additionally, these results indicate that higher concentrations of biocides (as found in PQ/MD and PQ/AD, containing 1.6 to 10 parts per million of each biocide) appear to be more efficacious at halting motility than those with lower concentrations of biocides (as found in PAPB/PQ and PHMB, containing 1 to 1.3 parts per million of each biocide). 

More critically, similar to previous studies noting that lenses and cases reduce CLC effectiveness due to biocide uptake [20,21], we noted in many places that in cycled PHMB (PHMB which has not been replaced after nightly use—the same aliquot of solution was used for nightly lens disinfection for 7 days, to simulate the real-world scenario of how many patients might misuse their CLCs), amoeba demonstrated significantly greater movement than in fresh PHMB. That is to say, cycled CLCs provided little to no disinfection efficacy, and were at many times not dissimilar from control solutions which contained no biocides. This further reinforces the vital need for physicians and product manufacturers to communicate to patients the importance of using their solutions appropriately. Finally, we demonstrated a relatively straightforward pattern of movement when displacement was compared to total and max distance traveled, during both the CLC and AC6 periods. This indicates that these amoebae were able to cover a considerable distance in any hour, as opposed to circling back on their own path, even after having been through a disinfection cycle. 

Following the conclusions gleaned from this study, it is important to note that some crucial examinations should be considered to continue answering the enquiries opened by these results. For instance, the scope of this study means that the relationship between motility and viability can only be inferred, although these results (including the percentage of moving amoebae during any one condition) do very closely resemble previously published viability data regarding these same CLC products and *Acanthamoeba* trophozoites [10,22]. In addition to correlations between motility and traditional viability studies (wherein amoebae are collected only at the end of the disinfection period and allowed to proliferate for two weeks before enumeration), crucial information could be gleaned by collecting viability data at hourly time points (similar to those used here) and conducting a more detailed time-based study. Furthermore, it is unknown how motility or speed affect infectivity, and paired in vivo studies would be required to truly correlate these parameters. 

## 5. Conclusions

In conclusion, we have estimated recovery after CLC disinfection by visualizing and tracking *Acanthamoeba* movement during and after CLC disinfection, using a previously described novel quantification method [8]. We have further demonstrated that solutions which rely on PHMB alone are less effective than other CLCs at halting *Acanthamoeba* motility, and that cycled CLCs are ineffective as disinfection agents. Most importantly, we demonstrated that *Acanthamoebae* are able to recover substantial motility following a disinfection phase, and surviving amoebae should still be considered motile. With these data, we conclude that it is critical that eyecare physicians recommend effective CLCs to their patients, and that patients are educated regarding, and adhere to, safe contact lens practices. 

## Figures and Tables

**Table 1:** Contact lens care (CLC) products used, their abbreviations, compositions, and manufacturer details.

Abbreviation	Composition	Product Name	Manufacturer
PQ/MD	polyquaternium-1 (0.001%), myristamidopropyl dimethylamine (0.0005%)	OPTI-FREE® Puremoist®	Alcon®, Fort Worth, TX, USA
PQ/AD	polyquaternium-1 (0.0003%), alexidine dihydrochloride (0.00016%)	ACUVUE® RevitaLens	Johnson & Johnson Vision Care, Jacksonville, FL, USA
PAPB/PQ	polyaminopropyl biguanide (0.00013%), polyquaternium (0.0001%)	Biotrue®	Bausch + Lomb®, Rochester, NY, USA
PHMB	polyhexanide (0.0001%)	Lite™	CooperVision®, San Ramon, CA, USA

## Data Availability

Please contact corresponding author regarding data.

## Supplementary Materials

The following supporting information can be downloaded at: https://www.mdpi.com/article/10.3390/microorganisms11020299/s1. Video S1. Example timelapse video of *Acanthamoeba* movement in experimental conditions during hour 18 (last hour of AC6 recovery), to demonstrate the differences in movement and possible recovery of amoebae after disinfection. Scale bar = 10mm. White arrows indicate remaining moving amoebae. Example calculations (using the representative videos in this figure) demonstrating the difference in using the average total distance moved in a frame vs. the relative units of net number of microns moved: One-quarter Ringer’s solution: Moving amoebae: 409; Average total distance of each amoeba: 457 µm; 409 amoebae × 457 µm = 186,913 µm net microns moved by amoebae in the field of view over one hour; PAPB/PQ: Moving amoebae: 33; Average total distance of each amoeba: 197 µm; 33 amoebae × 197 µm = 6501 µm net microns moved by amoebae in the field of view over one hour. Figure S1. Percentage of amoebae which are moving in any one field of view, with both strains combined. Amoebae were disinfected or put in one-quarter Ringer’s solution for the first 6 h, followed by AC6 media for the following 12 h (total 18 h). Shown in log scale to visualize differences between conditions. *n* = 3/group/strain. Figure S2. Data tables summarizing numerical findings at key time points, from Figure 4 and Figure 6. Amoebae were disinfected or put in one-quarter Ringer’s solution for the first 6 h, followed by AC6 media for the following 12 h (total 18 h). (**A**,**B**) Normalized total distance is presented as mean µm traveled per hour, multiplied by the number of moving amoebae per field of view. (**C**,**D**) Speed is presented as mean µm per second, multiplied by the number of moving amoebae per field of view (relative units). *n* = 3/group. Relevant standard error and statistical comparisons presented in Figure 4 and Figure 6.
