# Supplementary material for: Complete Recovery of Acanthamoeba Motility among Surviving Organisms after Contact Lens Care Disinfection
Source: Microorganisms. 2023 Jan 23;11(2):299. doi: 10.3390/microorganisms11020299 (PMC9965617; doi:10.3390/microorganisms11020299)
Supplement: Supplementary file 1 [file microorganisms-11-00299-s001.zip › microorganisms-2144684-supplementary.pdf]

Supplementary Materials: The following supporting information can be downloaded at: www.mdpi.com/xxx/s1**. Video S1.** Example timelapse video of *Acanthamoeba* movement in experimental conditions during hour 18 (last hour of AC6 recovery), to demonstrate the differences in movement and possible recovery of amoebae after disinfection. Scale bar = 10mm. White arrows indicate remaining moving amoebae. Example calculations (using the representative videos in this figure) demonstrating the difference in using the average total distance moved in a frame vs. the relative units of net number of microns moved:

One-quarter Ringer's solution:
Moving amoebae: 409
Average total distance of each amoeba: 457 µm
409 amoebae x 457 µm = 186,913 µm net microns moved by amoebae in the field of view over one hour

PAPB/PQ:
Moving amoebae: 33
Average total distance of each amoeba: 197 µm
33 amoebae x 197 µm = 6,501 µm net microns moved by amoebae in the field of view over one hour

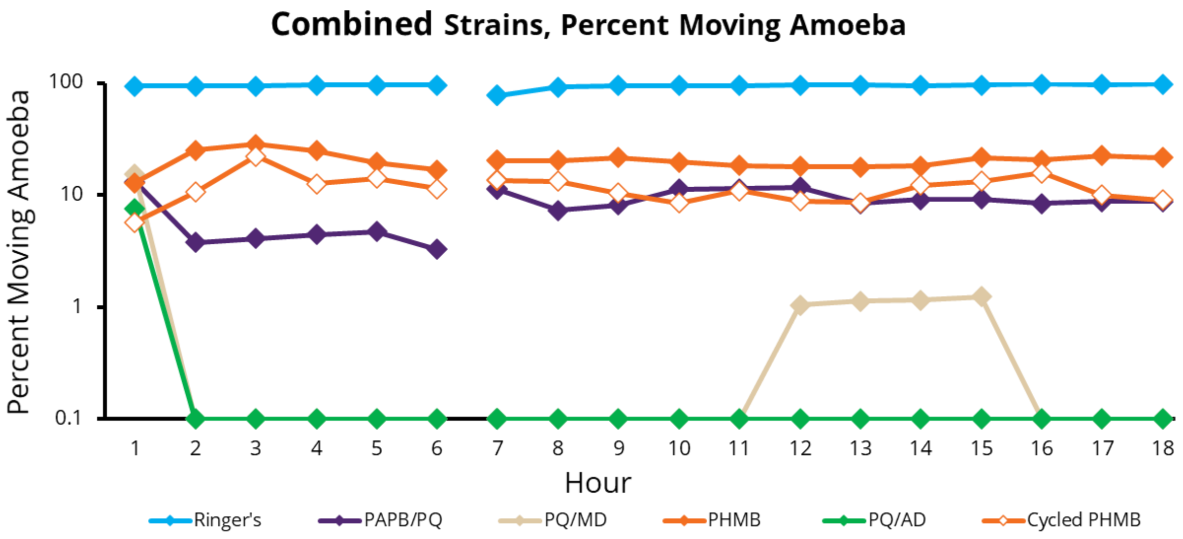

Figure S1. Percentage of amoebae which are moving in any one field of view, with both strains combined. Amoebae were disinfected or put in one-quarter Ringer's solution for the first 6 h, followed by AC6 media for the following 12 h (total 18 h). Shown in log scale to visualize differences between conditions. *n* = 3/group/strain.

A

Total Distance					
**50370**	**CLC**			**AC6**	
**Hour**	**1**	**3**	**6**	**12**	**18**
**Ringer's**	172182.1	143914.7	149368.3	140863.5	135344.6
**PHMB**	9402.6	36382.8	22693.2	49843.7	73428.4
**Cycled PHMB**	1129.2	39336.4	32226.1	44716.0	43024.0
**PAPB/PQ**	213.2	425.1	1689.2	7972.8	7824.5
**PQ/MD**	607.9	0.0	0.0	933.2	367.3
**PQ/AD**	138.4	0.0	0.0	0.0	0.0

B

Total Distance					
**30461**	**CLC**			**AC6**	
**Hour**	**1**	**3**	**6**	**12**	**18**
**Ringer's**	156380.5	140067.0	119209.2	98284.6	92958.2
**PHMB**	38924.3	51017.6	18116.2	26131.0	20736.1
**Cycled PHMB**	50081.2	91675.0	30811.4	9112.2	8384.0
**PAPB/PQ**	17124.9	1722.9	166.8	8001.7	1598.4
**PQ/MD**	17667.4	0.0	0.0	121.9	167.7
**PQ/AD**	5771.6	0.0	0.0	0.0	0.0

C

Speed					
**50370**	**CLC**			**AC6**	
**Hour**	**1**	**3**	**6**	**12**	**18**
**Ringer's**	47.9	40.2	41.7	39.4	45.2
**PHMB**	2.6	10.2	6.3	13.9	20.5
**Cycled PHMB**	0.3	11.0	9.0	12.5	12.0
**PAPB/PQ**	0.0	0.1	0.5	2.2	2.2
**PQ/MD**	0.2	0.0	0.0	0.3	0.1
**PQ/AD**	0.0	0.0	0.0	0.0	0.0

D

Speed					
**30461**	**CLC**			**AC6**	
**Hour**	**1**	**3**	**6**	**12**	**18**
**Ringer's**	43.5	39.1	33.3	27.4	24.1
**PHMB**	10.8	14.3	5.1	7.3	5.8
**Cycled PHMB**	13.9	25.6	8.6	2.6	2.3
**PAPB/PQ**	4.8	0.5	0.1	2.2	0.4
**PQ/MD**	4.9	0.0	0.0	0.0	0.1
**PQ/AD**	1.6	0.0	0.0	0.0	0.0

Figure S2. Data tables summarizing numerical findings at key time points, from Figures 4 and 6. Amoebae were disinfected or put in one-quarter Ringer's solution for the first 6 h, followed by AC6 media for the following 12 h (total 18 h). (**A**,**B**) Normalized total distance is presented as mean µm traveled per hour, multiplied by the number of moving amoebae per field of view. (**C**,**D**) Speed is presented as mean µm per second, multiplied by the number of moving amoebae per field of view (relative units). n = 3/group. Relevant standard error and statistical comparisons presented in Figures 4 and 6.
